# *Viburnum opulus* Fruit Phenolic Compounds as Cytoprotective Agents Able to Decrease Free Fatty Acids and Glucose Uptake by Caco-2 Cells

**DOI:** 10.3390/antiox8080262

**Published:** 2019-08-01

**Authors:** Małgorzata Zakłos-Szyda, Nina Pawlik, Dominika Polka, Adriana Nowak, Maria Koziołkiewicz, Anna Podsędek

**Affiliations:** 1 Institute of Technical Biochemistry, Faculty of Biotechnology and Food Sciences, Lodz University of Technology, Stefanowskiego 4/10, 90-924 Lodz, Poland; 2 Institute of Fermentation Technology and Microbiology, Faculty of Biotechnology and Food Sciences, Lodz University of Technology, Wólczańska 171/173, 90-924 Lodz, Poland

**Keywords:** *Viburnum opulus*, phenolic compounds, free fatty acids uptake, glucose uptake, cytoprotection

## Abstract

In recent years, there has been increasing interest in studying food-originated phytocompounds with beneficial influences for humans. Amongst the most active natural substances are polyphenols, for which high content has been identified in the *Viburnum opulus* berry, and which are unused in Western Europe. Due to its strong antioxidant activity we explored the potential of *V. opulus* as a preventive agent against diet-related chronic diseases, such as obesity and type 2 diabetes. Among the causes of these ailments is oxidative stress, as well as impaired glucose and free fatty acids (FFA) uptake. Thus, the purpose of this study was to determine biological activity of *V. opulus* phenolic extracts as cytoprotective agents able to decrease induced oxidative stress, lower lipid accumulation and attenuate glucose and FFA uptake by Caco-2 cells via GLUT2 and CD36/FAT transporters. To determine the source of the most biologically active phenolic compounds, we obtained four phenolic compounds extracts as crude juice, phenolics isolated from juice and two preparations of phenolics obtained with different extraction agents from fruit pomace. Among the studied extracts, the phenolic rich fraction obtained from fruit juice revealed the strongest activity to decrease uptake of glucose, FFA and accumulation of lipid droplets in Caco-2 cells without affecting their viability (IC_0_ 50 μg/mL). Observed uptake attenuation was followed by decrease of the *CD36/FAT* gene expression, without influence on the *GLUT2* and *PPARα* levels. We suspect that *V. opulus* phenolics were able to modulate the cellular membrane dynamic, although that hypothesis requires further, more detailed studies. Extracts revealed strong chemo-preventive activity against oxidative stress induced chemically by tert-butylhydroperoxide (t-BOOH), as well as against DNA damage through the induction of DNA repair after cell exposition to methylnitronitrosoguanidine (MNNG) and H_2_O_2_. Our findings suggest *Viburnum opulus* fruit as a dietary source of phytocompounds, which could be considered as a tailored design food supplement components for the prevention and treatment of postprandial elevation of glucose and fatty acids through delaying the rate of glucose and fatty acid absorption by intestinal cells.

## 1. Introduction

Recently, increased attention has been paid in research to food-originated phytocompounds with beneficial influences for humans. Amongst the most active natural substances are polyphenols, whose strong antioxidant activity is involved in the prevention and cure of many human ailments [1]. A high content of polyphenolic compounds has been identified in *Viburnum opulus* (guelder rose) berries, such as hydroxybenzoic acids, tannins, anthocyanins, chlorogenic acid, (+)-catechin, (−)-epicatechin, cyanidin-3 glucoside, cyanidin-3-rutinoside and quercetin [2,3]. Due to the antioxidant, antibacterial, hepatoprotective, high blood pressure-lowering and anti-inflammatory activities of *Viburnum*, these fruit have been used as a traditional medicine for treatment of ureteral stones, and cardiovascular or gastrointestinal diseases [4,5,6]. Whilst the plant is well known in Eastern Europe and Turkey (as gilaburu), where it can be found in food products such as juice, jams, jellies, marmalades and fermented drinks, in Western countries, as well as in Poland, is largely unused [7]. 

Our previous studies revealed that *V. opulus* fruit phenolic extract acted as an inhibitor of enzymes involved in lipid and carbohydrate metabolism, such as α-amylase and α-glucosidase, or PTP-1B phosphatase—protein, which is known as the major negative regulator in insulin and leptin signaling [8]. What is more, it possessed strong cytoprotective activity against oxidative stress induced chemically by tert-butylhydroperoxide (t-BOOH) in insulin secreting βTC3 pancreatic beta cells. These biological activities have the potential to use *V. opulus* fruit phenolics as a preventive agent against pandemic diet-related chronic diseases, such as obesity and type 2 diabetes. These health disorders are connected with metabolic syndrome, which in turn is accompanied by hyperglycemia, impaired glucose tolerance, postprandial lipemia, oxidative stress and hypertension as a result of glucose and free fatty acid (FFA) homeostasis impairment [9]. Obesity and type 2 diabetes development strongly involve glucose and FFA. These compounds are released in the digestive tract after food matrices digestion and are further absorbed by intestinal enterocytes mainly. Since polyphenols obtained from dietary sources gain high concentration in gut lumen, they are able to regulate biological activities of the cells in the intestine. Recent data proved that polyphenols are able to influence intestinal processes of glucose and FFA absorption, not only via inhibition of digestive enzymes, but also by attenuation of their uptake [10,11,12]. Some investigations suggested that polyphenolic compounds lower glucose absorption via influence on the sodium-dependent glucose transporter 1 (SGLT1), however, their stronger inhibition potential was identified in the case of the sodium-free glucose transporter known as GLUT2 [13]. Hence polyphenolic attenuation of glucose uptake and transport across the small intestine may be a potential control mechanism of glycemia. In the case of FFA they can be absorbed mainly via protein transporters known as fatty acid binding protein CD36 (CD36/FAT), the plasma membrane fatty acid binding protein (FABPPM), caveolin1 and the fatty acid transport protein 4 (FATP4) [14]. Increased lipid absorption leads to elevated intracellular lipid bodies formation. Additional to that mentioned above and strongly deepening cellular misfunction factor is increased production of reactive oxygen species. That state, in turn, leads to the inflammatory reaction appearance and cellular damage progression. Due to their chemical structure, polyphenolic compounds are effective radical scavengers and have important function antioxidant defense. 

Because intestinal cells are mostly influenced by large quantities of dietary compounds, as a cellular model in our study human epithelial Caco-2 cell line was chosen. These cells are commonly used for in vitro studies of chemicals’ biological activities, as well as in the mechanism of intestinal absorption. As a polyphenolic compound plant material we used *V. opulus* fruit. To determine the source of the most biologically active phenolic compounds we obtained four phenolic compounds extracts as crude juice, phenolics isolated from juice, and two preparations of phenolics obtained with different extraction agents from fruit pomace. The purpose of this preliminary study was to determine biological activity of *V. opulus* phenolic extracts as cytoprotective agents able to decrease induced oxidative stress, lower lipid accumulation, and attenuate glucose and FFA uptake by Caco-2 cells via GLUT2 and CD36/FAT transporters. 

## 2. Materials and Methods

### 2.1. Chemicals and Reagents

All chemicals used, if not stated otherwise, were obtained from Sigma-Aldrich (Steinheim, Germany). Acetonitrile (Merck, Darmstadt, Germany) and formic acid (Sigma-Aldrich) were hyper grade for LC-MS. Folin–Ciocalteu reagent was obtained from POCH (Gliwice, Poland). Reference compounds were obtained from Sigma-Aldrich (Steinheim, Germany) ((+)-catechin, (−)-epicatechin, rutin, gallic acid), Extrasynthese (Lyon, France) (chlorogenic acid, cyanidin 3-glucoside, quercetin 3-glucoside, isorhamnetin, and isorhamnetin 3-rutinoside) and Phytolab (Vestenbergsgreuth, Germany) (neochlorogenic acid, procyanidin B1, and procyanidin B2). Ultrapure water (Simplicity^®^ Water Purification System, Millipore, Marlborough, MA, USA) was used to prepare all solutions. All cell culture reagents were obtained from Life Technologies (Carlsbad, CA, USA). Tissue culture plastics were supplied by Greiner Bio-One GmbH (Frickenhausen, Austria). All the experimental measurements, if not stated otherwise, were performed using the Synergy 2 BioTek Microplate Reader (Winooski, VT, USA). Microscopic observations were performed using fluorescent microscope Nikon TS100 Eclipse (Tokyo, Japan) under 200× magnification, if not stated otherwise. 

### 2.2. Preparation of Viburnum Opulus Extracts

**Figure 1:**
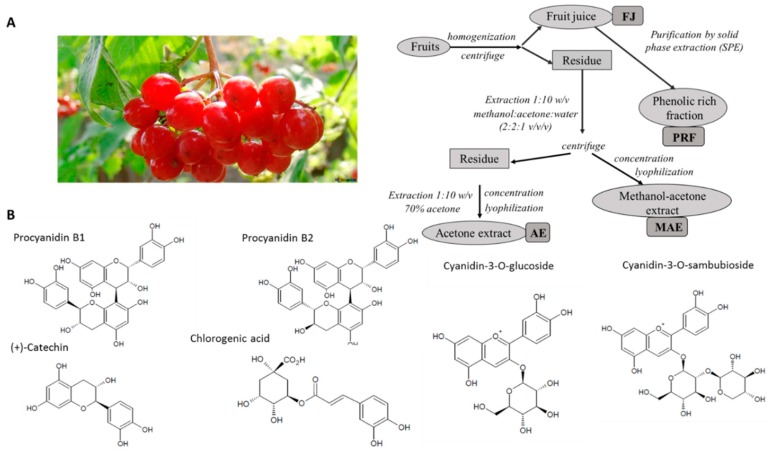
*Viburnum opulus* fruit as source of phenolic compounds. (**A**) Schematic diagram of *V. opulus* fruit samples preparation: FJ—juice, PRF—phenolic rich fraction, AE—acetone extract, MAE—methanol-acetone extract; (**B**) Structures of main phenolic compounds identified in fruit extracts.

Fresh *Viburnum opulus* L. fruits were obtained from Rogów Arboretum, Warsaw University of Life Sciences (Rogów, Poland) with the acc. no. 18162 identification. Berries were homogenized and the pulp was centrifuged at 5000 rpm for 10 min to obtain fresh juice (FJ) (Figure 1A). The solid residue was extracted with a mixture of methanol:acetone:water (2:2:1 *v*/*v*/*v*) in a ratio of 1:10 *w*/*v* on the stirrer at 800 rpm for 30 min. The slurry was centrifuged at 5000 rpm for 10 min. The solid residue was back-extracted with a 70% (*v*/*v*) acetone solution under the same conditions as the previous extraction. The supernatants were concentrated at 40 °C (vacuum rotary evaporator RII, Büchi, Switzerland) to remove organic solvent. Water concentrates were freeze-dried to afford methanol-acetone extract (MAE) and acetone extract (AE). The part of FJ was purified by solid-phase extraction using 12-Port Vacuum Manifold system. The C-18 Sep-Pak cartridge (10 g capacity, Waters Corp., Milford, MA, USA) was pre-treated with successive applications of 40 mL of methanol and 40 mL of water and the FJ (2 mL) was passed through the column. Phenolic compounds were bound to the C-18 cartridge, while sugars and other polar compounds were removed with water (40 mL). Then, phenolic compounds were eluted with methanol (40 mL). After methanol removing under reduced pressure (T < 40 °C), solid residue was dissolved in water and lyophilized to afford phenolic-rich fraction (PRF). For biological activity assays MAE, AE and PRF were dissolved in a water/dimethyl sulfoxide (DMSO) (1:1 *v*/*v*) at concentration 100 mg/mL.

### 2.3. Determination of Total Phenolics Content and Phenolic Compounds Profile

The content of the total phenols was determined by using the Folin–Ciocalteu method [8,15]. The results were calculated as mg of gallic acid equivalent (GAE) in 1 g of extract or juice. Phenolic profiles were determined using an ACQUITY Ultra Performance LC system (UPLC) equipped with a photodiode array detector with a binary solvent manager (Waters, Milford, MA, USA). The data was collected by Mass-Lynx™ V 4.1 software. Separation was achieved on a Acquity UPLC HSS T3 column (150 × 2.1 mm, 1.8 µm; Waters). The mobile phase was a binary gradient with A, water/formic acid (95.5:4.5, *v*/*v*), and B, acetonitrile, with a flow rate of 0.45 mL/min. The binary gradient was as follows: initial conditions—99% A (0 min), 12 min—75% A, 12.5 min—100% B, —99% A (12.5–13.5 min). The runs were monitored at the following wavelengths: for flavanols and hydroxybenzoic acids at 280 nm, hydroxycinnamic acids at 320 nm, flavonols at 360 nm and anthocyanins at 520 nm. The retention times and spectra were compared to those of the authentic standards. Calibration curves at concentrations ranging from 0.06 to 2 mg/mL (r ≥ 0.96) were made from chlorogenic acid, gallic acid, neochlorogenic acid, (+)catechin, (−)epicatechin, procyanidins B1 and B2, cyanidin 3-glucoside, cyanidin 3-rutinoside, cyanidin 3-sambubioside, quercetin 3-glucoside, rutin, isorhamnetin, and isorhamnetin 3-rutinoside. The results are expressed as mg of phenolic compounds per g of freeze dried preparation of extract or juice.

### 2.4. Antioxidant Capacity Assays 

The antioxidant capacity of different *V. opulus* (VP) berry samples was determined by reduction of the ABTS (2,2’-azino-bis(3-ethylbenzothiazoline-6-sulphonic acid)) free radical cation described previously [8]. The results were expressed as TEAC (trolox equivalent antioxidant capacity) calculated as µmol of trolox equivalent (TE)/1 g of sample tested. To perform ORAC (Oxygen Radical Absorbance Capacity) assay, AAPH (2,2’-Azobis(2-amidinopropane) dihydrochloride) was used as a peroxyl radical generator, fluorescein as a fluorescent probe and trolox as a standard. All reagents were prepared in 75 mM phosphate buffer, pH 7.4. All experimental wells contained 150 μL fluorescein (4 μM) and 25 μL of diluted sample and were pre-incubated for 15 min at 37 °C before injection of 25 μL AAPH solution (173 mM). The fluorescent signal at F485/520 nm was measured every 2 min for 2 h. Final fluorescence measurements were expressed relative to the initial reading. Results were calculated based on differences of areas under the fluorescein decay curve (AUC) between the blank and a sample. The antioxidant capacity was expressed as µmol of trolox equivalent (TE) per gram of sample tested.

### 2.5. Cell Culture and Exposure Conditions

Human colon adenocarcinoma cell line Caco-2 cell line was obtained from ATCC, Lot Number: 58844056, ampule passage number 18. Cells were grown in DMEM with 10% fetal bovine serum (FBS) medium supplemented with 100 U/mL penicillin, 100 μg/mL streptomycin and 25 μg/mL amphotericin B. The cells were maintained at 37 °C in a humidified incubator containing 5% CO_2_. Cells were incubated with tested extracts for 24 h. Tested MAE, AE and PRF lyophilizates were dissolved in a water/DMSO (1:1 *v*/*v*) at concentration 100 mg/mL and were further diluted with culture medium. The extract’s concentrations used in biological studies are presented in the descriptions of the tests carried out.

### 2.6. Cell Viability

Cells were seeded into 96-well plates at 10^4^ cells per well in complete medium and grown for 20h, then incubated in the presence of the studied extracts diluted in DMEM culture medium for either 24 h. Cell viability was quantified with PrestoBlue reagent according to the manufacturer’s instructions by measuring the fluorescent signal at F530/590 nm. The obtained fluorescence values were used to calculate cell viability expressed as the percentage of the viability of the untreated control cells (cells treated with equal volume of the vehicle instead of the preparation). To evaluate the protective effect of preparations against oxidative stress cells were pre-incubated with IC_0_ of extracts for 24 h [16]. Then, to induce the oxidative stress condition, 500 μM t-BOOH was added for 2 h and cellular viability with PrestoBlue reagent was measured.

### 2.7. Detection of Intracellular Reactive Oxygen Species (ROS) Generation

To determine the effect of extracts on the intracellular generation of ROS after 24 h treatment with extracts, cells were loaded with the DCFH-DA (dichloro-dihydro-fluorescein diacetate) dye at a final concentration of 10 μM for 30 min. Fluorescent signal at F485/530 nm was analyzed. For microscopic observations, the experiment was conducted in 8-well Lab-Tek™ Chamber Slides. The negative control contained only cells in DMEM (without FBS), while positive control contained 500 µM t-BOOH. The intracellular fluorescence of cells was observed after cells treatment with chemicals under fluorescence microscope. Increased intensity of intracellular fluorescence was indicative of elevated level of generated ROS.

### 2.8. Measurement of ATP Production

The intracellular ATP (adenosine triphosphate) level was quantified with CellTiter-Glo^®^ Luminescent Cell Viability Assay. Briefly, after cells incubation with the extracts for 24 h the single reagent was added directly to the cells; after cells lysis luminescent signal proportional to the amount of ATP present was generated and measured. 

### 2.9. Measurement of Mitochondrial Membrane Potential (MMP)

The MMP was assayed with JC-1 probe. After 24 h treatments with the studied extracts the medium was changed and JC-1 (1 μg/mL) was added for 20 min [17]. Then, the cells were washed with serum-free medium and fluorescent signal at F485/530 nm was measured.

### 2.10. Phosphatidylserine Externalization, Membrane Permeabilization and Apoptosis Induction

To quantify the level of externalized phosphatidylserine on the outer membrane leaflet of apoptotic cells Annexin-V-FITC assay kit was used (Sigma-Aldrich). After 24 h treatment with extracts cells were washed twice with PBS (phosphate buffer saline) and incubated with annexin-V-FITC (final concentration 0.25 μg/mL) for 10 min. Annexin-V binding was measured by the change in fluorescence F485/530 nm. Membrane permeabilization caused by investigated compounds was measured with propidium iodide (PI). After cells treatment with extracts for 24 h PI was added at a final concentration of 1 µg/mL. Intercalation of PI was monitored by the change of fluorescence F535/620 nm. For intracellular Ca^2+^ measurement with the Screen Quest™ Fluo-8 No Wash Calcium Assay Kit (AAT Bioquest, Inc., Sunnyvale, CA, USA) was performed simultaneously according to the supplier’s protocol. The late stage of apoptosis was measured by Cell Death Detection ELISA Plus (Roche, Basel, Switzerland) according to manufacturer’s instructions. After 24 h treatment cells were lysed and histone-complexed DNA fragments (mono- and oligonucleosomes) present in the cytoplasmic fraction were quantified with immunoreagent complex. DNA-histone complex served as the positive control (PC). Following incubation and washes, the colorimetric solution was added and after adding the stop solution the colorimetric signal was measured at 405 and 490 nm. Calculation of enrichment factor of mono- and oligonucleosomes released into the cytoplasm was performed according to the formula:

enrichment factor [%]=absorbance of the sample cells/absorbance of the control cells ×100

In order to detect necrosis after cells incubation with compounds medium was collected and the level of DNA fragments released from necrotic cells was determined analogously to the apoptosis measurement. 

### 2.11. Glucose Uptake

After incubating the cells for 24 h with the preparations 150 μM 2-NBDG (2-(N-(7-nitrobenz-2-oxa-1,3-diazol-4-yl)amino)-2-deoxyglucose) was added in the glucose- and serum-free medium. After 2 h of incubation with fluorescent glucose analogue cells were washed twice with serum- and glucose-free medium and fluorescent signal at F485/530 nm was measured immediately [17].

### 2.12. Nile Red Staining 

Cells were incubated in FBS-free medium for 24 h with the extracts in the presence of 100 μM palmitic acid. After treatment, cells were washed with cold PBS, fixed in 5% paraformaldehyde for 30 min and stained with Nile red solution (1 μg/mL) for 40 min [18]. The lipid-bound Nile red fluorescent signal F485/530 was measured. 

### 2.13. Fatty Acid Uptake

After incubating the cells for 24 h with the preparations TF2-C12 stock solution was added to serum- and glucose-free medium according to the manufacturer’s procedure (Fatty Acid Uptake Kit, Sigma-Aldrich). After 60 min incubation with cells fluorescent signal at F485/530 nm was measured. 

### 2.14. DNA Damage and Repair

Extracts were investigated in terms of their ability to induce DNA repair in Caco-2 cells that had been exposed to two mutagens: MNNG (methylnitronitrosoguanidine), and hydrogen peroxide. Briefly, cells were damaged with MNNG (6.8 μM) or hydrogen peroxide (25 μM) for 10 min on ice. After centrifugation (182× *g*, 4 °C, 15 min) cells were exposed for 60 and 120 min to the extracts at IC_0_, sampled cells’ DNA repair activity was stopped in an ice bath. At each time interval DNA repair was quantified by determination of the extent of residual DNA damage. The positive controls were exposed only to MNNG or hydrogen peroxide, while the negative control consisted of cells in DMEM. The alkaline comet assay was performed as previously described [19]. Slides after neutralization were stained with DAPI (1 mg/mL) and visualised at 200× magnification in a fluorescence microscope (Nikon Eclipse Ci H600L, Tokyo, Japan). Fifty images were randomly selected from each sample and the percentage of DNA in the comet tail was measured.

### 2.15. Gene Expression Analysis

Total RNA was extracted from Caco-2 cell culture after 24 h incubation with extracts using RNeasy^®^ Mini Kit (QIAGEN, Venlo, The Netherlands) and purified with Amplification Grade DNase I. RNA samples were reverse transcribed with RT^2^ First Strand Kit (SABioscences, Frederick, MD, USA). Gene expression was normalized using constitutively expressed hypoxanthine phosphoribosyltransferase 1 (*HPRT1*) as a reference gene. The following primer sequences were used to determine the genes’ expression: *GLUT2*, forward primer TTGAAGCCACAGGTTGCTGA and reverse primer CCTGCCTGTTTATTCGCAGC; *FAT/CD36*, forward primer AGTTGGAACAGAGGCTGACAACT and reverse primer TATGGGATGCAGCTGCCCACACG; *PPARα*, forward primer TCCTGAGCCATGCAGAATTTAC and reverse primer AGTCTAAGGCCTCGCTGGTG; *HGPRT1*, forward primer TGACCAGTCAACAGGGGACA and reverse primer AAGCTTGCGACCTTGACCAT. Real time RT-PCR was carried out using SYBR^®^ Greenbased RT2 qPCR Master Mix (SABioscences) on a BioRad CFX96 qPCR System (Bio-Rad, Hercules, CA, USA). Complementary DNA representing 6 ng of total RNA per sample was subjected to 40 cycles of PCR amplification. Samples were first incubated at 95 °C for 40 s, then at 58 °C for 30 s, and finally at 72 °C for 30 s. To exclude non-specific products and primer-dimers, after the cycling protocol, a melting curve analysis was performed by maintaining the temperature at 52 °C for 2 s, followed by a gradual temperature increase to 95 °C. The threshold cycle (Ct) values for that gene did not change in independently performed experiments. The level of target gene expression level was calculated as 2^−ΔΔCt^, where ΔΔCt = [Ct(target) − Ct(GAPDH)]sample − [Ct(target) − Ct(GAPDH] calibrator.

### 2.16. Statistical Analysis

All data are presented as mean ± S.D. from at least three independent experiments. All obtained results were subjected to statistical analysis. Composition data was analyzed by means of a one-way ANOVA using Statistica Ver. 6.0 (TIBCO Software Inc., Palo Alto, CA, USA), and Duncan’s post hoc test was used to assess the differences between the means with significance level *p* ≤ 0.05. Pearson’s correlation coefficients were determined using Microsoft Excel XP. For biological studies determination of average values and one-way ANOVA analysis followed by the Dunnett’s test were performed using GraphPad Prism 6.0 software (GraphPad Software, Inc., La Jolla, CA, USA) at the significance level of * *p* ≤ 0.05, ** *p* ≤ 0.01, *** *p* ≤ 0.001. For DNA damage and repair results two-way analysis of variance (ANOVA) was conducted using OriginPro 6.1 software to evaluate the experimental data. 

## 3. Results and Discussion

### 3.1. Phenolic Compounds Profile and Content

Phenolic composition of FJ, PRF, MAE and AE determined by UPLC method is shown in Table 1. Based on a comparison of retention times and UV-vis spectra (200–600 nm) with the data for standards in the tested samples, 14 phenolic compounds have been marked with the significant differences (*p* ≤ 0.05) in the content of individual phenolic compounds. Among samples tested, the FJ extract contained the lowest level of phenolic compounds: due to the low content of dry matter (8.51%) phenolics accounted for 1.03% of the weight of the juice. However, beside phenolic compounds, the juice contained sugars, proteins, organic acids and different mineral ingredients. The highest concentrations of individual phenolic compounds were determined in PRF obtained from FJ and reached value 827.00 mg/g of preparation (82.7%). Thus, the FJ purification process on Sep-Pac C18 column resulted in an 80-fold increase in the content of phenolic compounds. Our research confirmed that *V. opulus* berry pomace is a valuable source of natural antioxidants because the extracts obtained from fruit pomace using two different extraction solvents contained 10.17% (AE) and 12.17% (MAE) of phenolic compounds. In all samples tested, the dominant phenol components were hydroxycinnamic acids. They accounted for about 77%, 78%, 79% and 83% of the sum of the phenolics determined in FJ, PRF, MAE and AE, respectively. Chlorogenic acid was identified as the main phenolic compound with the highest content in PRF, which exceeded in this respect about 7-, 8- and 81-times MAE, AE and FJ, respectively. Chlorogenic acid has been described previously as the main phenolic component in *V. opulus* fruit and juice, where it constitutes more than 96.2% of dihydroxycinnamic acid derivatives [3]. This phenolic acid is known for its numerous biological activities both in vitro and in vivo, i.e., antioxidant, antimicrobial and anti-inflammatory properties [20]. Quantitatively, flavanols were the second component of the *V. opulus* samples tested with (+) catechin as the main ingredient. Its concentration varied from 1.21 mg/g of FJ to 100.74 mg/g of PRF. ACE and ME also contained procyanidins B1 and B2, and (−)-epicatechin, whereas the latter has not been determined in FJ and PRF. Flavanols accounted for about 12%, 19%, 15% and 12% of total phenolics determined in FJ, MAE, AE and PRF, respectively. In *V. opulus* fruit this group of phenolic compounds accounted for 14% of the sum of the phenolics determined by HPLC method [21]. In the present study, flavonols with content in the range of 0.01–2.35 mg/g occurred at the lowest concentration in all samples with qualitative profile varying depending on the sample. For example, MAE did not contain rutin and isorhamnetin, while AE isorhamnetin, and FJ did not contain quercetin-3-glucoside. In addition to the flavonols designated in our work, quercetin-pentose, quercetin-hexose, quercetin-deoxyhexose and isorhamnetin glycosides have been found in other research [3,22]. There have been identified ten different cyanidin glycosides in the fruit, however, we have determined the total content of three of these in the range 1.12–78.06 mg/g of the sample. Cyanidin 3-sambubioside was the main pigment in FJ and PRF while cyanidin 3-glucoside dominated in extracts obtained from pomace (MAE and AE). 

The total phenolic compounds’ content as gallic acid equivalents (GAE) was determined by spectrophotometric method with Folin–Ciocalteu reagent and is presented in Table 2. The results obtained were congruent with the results achieved by the UPLC method with the decreasing rank of total phenolics content as follows: PRF > MAE > AE > FJ. The presented results are consistent with other reports with concentration of total phenolics in *V. opulus* juice between 5.47 and 7.78 mg GAE/g, as well as 70.99 mg GAE/g dw berries [8,23]. 

### 3.2. Antioxidant Capacity of V. opulus Extracts

The content of phenolic compounds in plant-derived products is associated with their antioxidant properties, which in turn is related to health benefits. Antioxidant properties of *V. opulus* samples tested were estimated as scavenging potential toward ABTS•+ and peroxyl radical (ROO•) cations and were expressed as trolox equivalents (TE). However, the ABTS•+ method is based on the use of synthetic radical cation not found in the biological system, it is still widely used in the study of the antioxidant capacity of pure compounds and complex extracts. The other reference method used for the determination of antioxidant activity, especially in food analysis, is ORAC. The main advantage of the ORAC method is its ability to analyze mixtures containing various antioxidants with varying antioxidant activity, as well as components that do not exhibit antioxidant properties. The disadvantage of that chemical method of determining the antioxidant capacity is the deficiency of representation of antioxidant capacity in the biological system. The total antioxidant capacity values were found between 38.36 and 2619.59 µmol TE/g in the ABTS method, and from 103.50 to 7810.29 µmol TE/g in the ORAC method (Table 2). The highest antioxidant capacity characterized PRF, while the lowest activity had FJ. The higher phenolic content in a sample showing greater scavenging activity against free radicals was confirmed by high Pearson’s correlation coefficients (r = 0.993 and 0.991 between total phenolics, and ABTS and ORAC values, respectively). Despite the differences in antioxidant capacities obtained with these methods, which result from different chemical structures of radicals used, the potential of extracts’ scavenging activities was analogous. For comparison, the antioxidant capacity of *V. opulus* fruit varied from 127.37 to 189.98 µmol TE/g in the ORAC method [23]. 

### 3.3. Assessment of Viburnum Opulus Phenolics on Cellular Viability 

**Figure 2:**
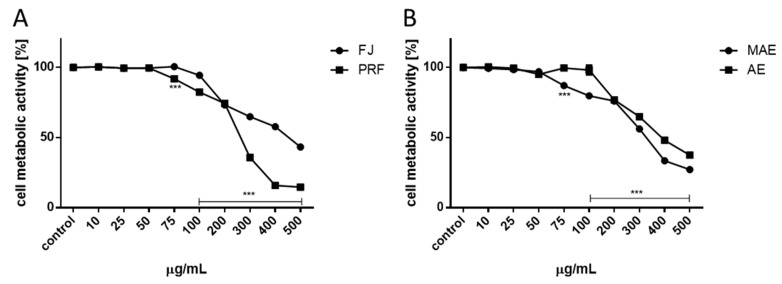
The influence of *V. opulus* extracts on Caco-2 cells on metabolic activity determined by PrestoBlue assay after 24 h exposure of juice (FJ) and PRF (phenolic rich fraction) (**A**), MAE (methanol-acetone extract) and AE (acetone extract) (**B**). Control cells were not exposed to any compound but the vehicle; values are means ± standard deviations from at least three independent experiments; statistical significance was calculated versus control cells (untreated) *** *p* ≤ 0.001.

First the effect of extracts’ concentrations from 10 to 500 μg/mL (µg of freeze-dried extract/mL) on cellular viability was studied after 24 h incubation. As is presented in Figure 2A,B, the metabolic activity decreased with increasing extract concentrations starting from 50 μg/mL. The highest toxicity against cells revealed PRF, where 300 μg/mL dosage decreased cellular activity to 36.5% compared to the control cells. Fresh juice had the least influence on metabolic activity: the same dosage decreased cells viability to 65.5%, whilst the highest concentration tested (500 μg/mL) was able to inhibit cells viability to 43.5%. That extract contained the smallest amount of phenolics in dry mass. The highest noncytotoxic concentrations (IC_0_) selected for further studies, as well as IC_50_ values (the chemical concentration required to reduce the cell activity to 50% of the control), are summarized in Table 3. According to IC_50_ values, the cytotoxicity of *V. opulus* extracts ranked as follows: PRF > MAE > AE > FJ. The IC_0_ concentration used in this work (50–100 µg/mL) can be achieved in the gut under physiological conditions [24]. 

### 3.4. Viburnum opulus as a Chemopreventive Agent 

**Figure 3:**
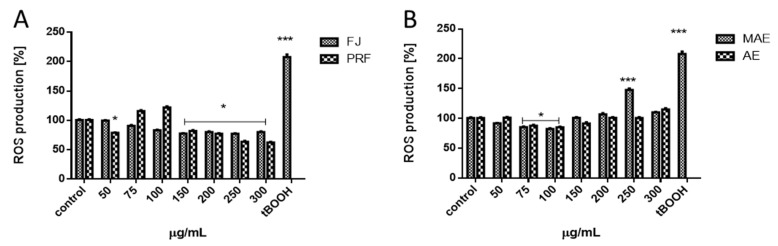
The effect of *V. opulus* extracts on intracellular ROS generation analyzed by DCFH-DA assay after 24 h incubation with juice (FJ) and PRF (phenolic rich fraction) (**A**), MAE (methanol-acetone extract) and AE (acetone extract) (**B**). Control cells were not exposed to any compound but the vehicle; values are means ± standard deviations from at least three independent experiments; statistical significance was calculated versus control cells (untreated) * *p* ≤ 0.05, *** *p* ≤ 0.001.

It is known that chronic glucose and free fatty acids elevation or chronic hypoxia lead to excessive oxidative stress defined as an imbalance between removal and formation of ROS [25]. Its consequences are insulin resistance generation, metabolic failure and finally cells death. Cells pre-incubation with extracts at IC_0_ decreased intracellular ROS level by 20% compared to the cells treated with the vehicle only (Figure 3A,B). *V. opulus* cytoprotective activity has a similar pattern as results obtained using chemical assays: the strongest potential revealed PRF, while the lowest FJ. However, cellular antioxidant capacity using a reduced DCF probe takes into account the bioavailability, distribution and metabolism of antioxidants within the cell and reflects the activity of the tested extracts in the biological system.

**Figure 4:**
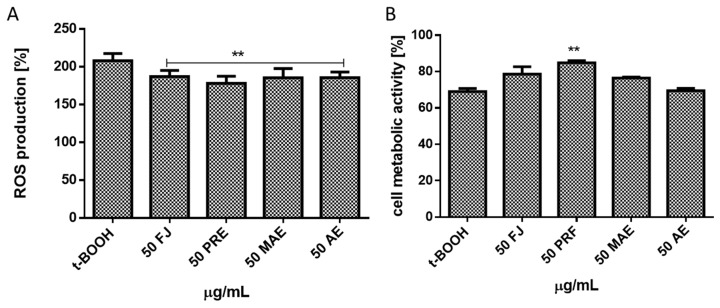
The cytoprotective properties of *V. opulus* extracts (FJ—fresh juice, PRF—phenolic rich fraction, MAE—methanol-acetone extract, AE—acetone extract) at 50 µg/mL dosage (24 h preincubation) against chemically induced (500 µM t-BOOH, 2 h) oxidative stress analyzed with DCFH-DA (**A**) and on metabolic activity analyzed with PrestoBlue assay (**B**). Control cells were not exposed to any compound but the vehicle; values are means ± standard deviations from at least three independent experiments; statistical significance was calculated versus control cells (untreated) ** *p* ≤ 0.01.

To evaluate *V. opulus* chemopreventive properties we treated Caco-2 cells with tert-butylhydroperoxide (t-BOOH), a strong pro-oxidant and in vitro oxidative stress inducer. As products of its activity appear tertbutoxyl, peroxyl, alkoxyl and methyl radicals catalyzing lipid peroxidation, DNA strand breaks and alteration in intracellular calcium homeostasis [26]. As compared to the untreated cells, t-BOOH lowered cells’ metabolic activity by almost 20% (Table 3) and significantly increased the intracellular ROS production (Figure 3A,B). However, cells pre-incubation with the preparations at IC_0_ dosages before t-BOOH treatment resulted in reduced t-BOOH induced oxidative stress by nearly 15%–20%, revealing their protective abilities (Figure 4A). Additionally, extracts were able to diminish the toxic effect of t-BOOH on metabolic activity (Figure 4B). The strongest protective properties revealed a polyphenolic rich fraction. These results confirm our previous data, where βTC3 cells pre-incubation with guelder rose fruit extract at 0.365 mg/mL protected cells against oxidative damage induced by t-BOOH [8]. We suppose that *V. opulus* polyphenols, due to their scavenging potential towards free radical cations, could protect the cellular membrane against oxidation via its stiffening, which in result hinders the spreading of newly generated radicals. It was shown that quercetin had beneficial effects on cell membranes’ lipid peroxidation by restoring membrane stiffening or decrease membrane fluidity [27]. Alternatively, phenolic compounds are known to enhance the detoxifying enzymes through evoking activity or recovery of glutathione (GSH)-S-transferase (GST), NADPH: quinone oxidoreductase 1, haem oxygenase 1 (HO1) and glutamyl-cysteine ligase (GSL) [28,29]. Studies performed on Ehrlich ascites carcinoma (EAC)-bearing mice demonstrated that *V. opulus* juice treatment was able to enhance activity of superoxide dismutase (SOD) and catalase (CAT) in liver and kidney [6]. Furthermore, polyphenols interactions with membrane-localized lipid rafts may influence synthesis and activity of the nuclear transcription factor Nrf2, which is known as the master regulator of cellular resistance to oxidative stress [30]. In the case of chlorogenic acid, its ability to change lipid packing order in the hydrophilic part of the membrane without influence on membrane fluidity was demonstrated [31]. Nonetheless, chlorogenic acid and its isomers were revealed as modulators of redox biology in inflamed Caco-2 via Nrf2 signaling by nuclear translocation of Nrf2 and upregulation of Nrf2 gene expression [30,32]. That was accompanied by reduction of inflammatory interleukin-8 (IL-8) secretion by Caco-2 cells. 

**Figure 5:**
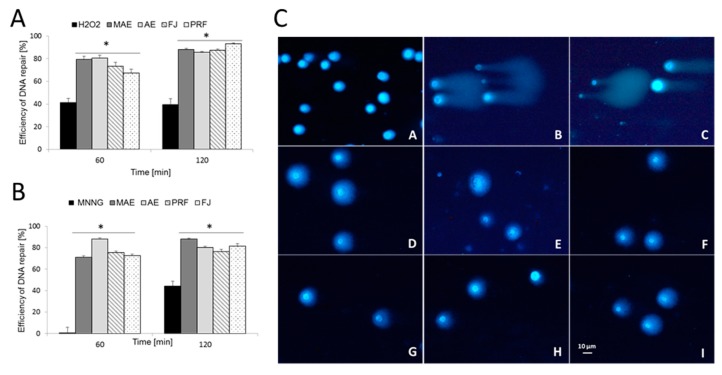
The *V. opulus* extracts’ (FJ—fresh juice, PRF—phenolic rich fraction, MAE—methanol-acetone extract, AE—acetone extract) activity as protectants against DNA damage. Efficiency of DNA repair (%) in Caco-2 cells exposed for 10 min to mutagens: (**A**) hydrogen peroxide (25 µM) and (**B**) MNNG (6.8 µM) on ice. The cells were then post-incubated (at 37 °C for 60 and 120 min) with extracts and DNA repair was measured in the time intervals. The number of cells analyzed for each time-interval was 50. Error bars denote S.E.M. ANOVA (*p* ≤ 0.05). * Significantly different from the positive control. Typical images of Caco-2 comets stained with DAPI: (*A*) negative control; (*B*) hydrogen peroxide (25 µM); (*C*) MNNG (6.8 µM). After pre-incubation with hydrogen peroxide, 120 min post-treatment with extracts: (*D*) MAE extract; (*E*) AE extract and (*F*) PRF extract. After pre-incubation with MNNG, 120 min post-treatment with extracts: (*G*) MAE extract; (*H*) AE extract and (*I*) FJ extract. Fluorescence microscopy (Nikon, Tokyo, Japan); 200× magnification (**C**).

Due to the fact that epithelial intestinal cells are strongly affected by mutagenic compounds, we have checked *V. opulus* activity as protectants against DNA damage through the induction of DNA repair with a comet assay (Figure 5A–C). In the experiment, cells were first challenged with mutagens (MNNG or hydrogen peroxide), which then were washed out and cells were post-incubated with extracts at IC_0_ dosages. In the case of the H_2_O_2_ positive control, which was not treated with plant extracts, approx. 40% efficiency of DNA repair was observed after 60 and 120 min in comparison to the initial point. For MNNG, the efficiency was observed only after 120 min, and it was approx. 45%. The effect was enhanced over 2 h and, after that time, DNA damage was approx. 40% lower than for the positive control at the same time point. Extracts induced DNA repairs more efficiently after exposition to hydrogen peroxide than MNNG reaching up to 93% and 88%, respectively. Cells exposed to PRF removed DNA breaks with the highest efficiency of 93% after 120 min after pre-treatment with hydrogen peroxide. MAE extract was found to be the strongest inducer of DNA repair in cells pre-treated with MNNG after 120 min and the efficacy was 88%. This is the first study showing the protective activity of *V. opulus* berries on human cells with respect to selected mutagens. In both preparations, chlorogenic acid dominates, which was identified as a preventive agent against isoproterenol-induced DNA damage [33]. 

### 3.5. Effect of V. opulus Extracts on Intracellular ATP Level, Intracellular Oxidative Stress and Apoptosis Induction

**Figure 6:**
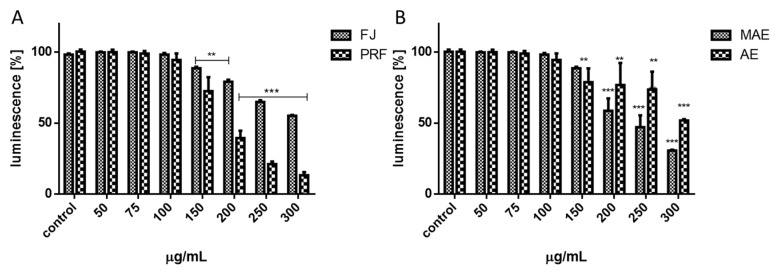
The influence of 24 h of exposure of *V. opulus* juice (FJ) and PRF (phenolic rich fraction) (**A**), MAE (methanol-acetone extract) and AE (acetone extract) (**B**) on ATP level in Caco-2 cells determined by ATP luminescent assay. Control cells were not exposed to any compound but the vehicle; values are means ± standard deviations from at least three independent experiments; statistical significance was calculated versus control cells (untreated), ** *p* ≤ 0.01, *** *p* ≤ 0.001.

**Figure 7:**
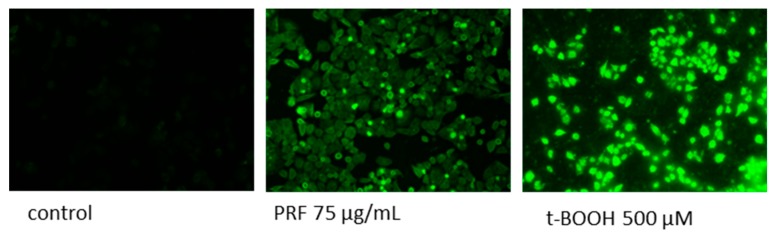
Intracellular ROS generation in cells after staining with DCFH-DA; cells were treated with PRF-phenolic rich fraction for 24 h; low fluorescence in control cells, very high fluorescence in positive control cells after incubation with 500 µM t-BOOH; fluorescence microscopy (Nikon TS100 Eclipse, Japan), 200× magnification. Images are representative of one of three similar experiments.

**Figure 8:**
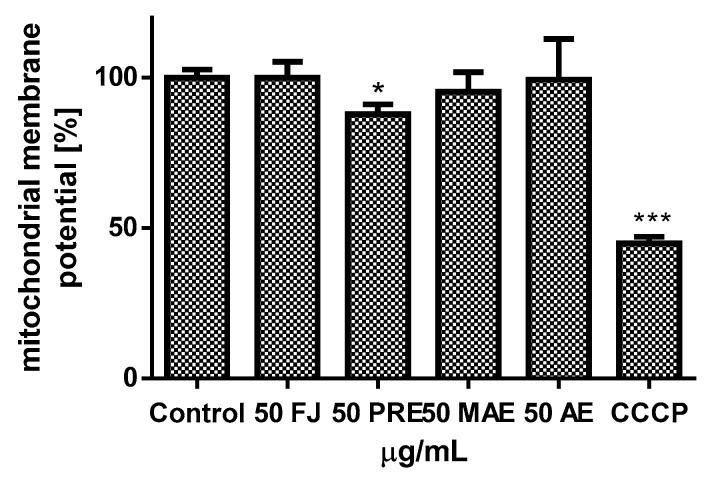
The *V. opulus* juice (FJ), PRF (phenolic rich fraction) MAE (methanol-acetone extract) and AE (acetone extract) (at 50 µg/mL dosage) influence on mitochondrial membrane potential was determined with JC-1 probe. As a positive control for depolarization, carbonyl cyanide m-chlorophenyl hydrazine (CCCP ) (50 μM) was used; control cells were not exposed to any compound but the vehicle; values are means ± standard deviations from at least three independent experiments; statistical significance was calculated versus control cells (untreated) * *p* ≤ 0.05, *** *p* ≤ 0.001.

Experimental data presented in Figure 6A,B confirmed that the values of extracts at concentrations close to calculated IC_50_ influenced cellular ATP production. After cells’ treatment with the FJ at 200 μg/mL, the ATP level decreased by 20%, and that effect deepened to 50% at the highest concentration (300 μg/mL). At comparable concentrations, PRF reduced luminescence values by 30% and 85%, respectively. The components of MAE and AE obtained from solid residue lowered the ATP level to 85% and 76% at 150 μg/mL dosage, respectively. As was previously shown, we observed the intracellular increase of reactive oxygen species level: PRF was the strongest oxidative stress inducer and at 75 μg/mL dosage elevated fluorescence by nearly 20% in comparison to control. That quantitative result has been confirmed by fluorescent microscopic observations where cells treated with PRF after staining with fluorogenic dye DCFH-DA revealed higher fluorescence (DCF-DA is oxidized by ROS into 2,7-dichlorofluorescein) compared to the untreated cells, due to the higher ROS concentration (Figure 7). Surprisingly, under the same conditions of experiment we observed the 15% decrease of MMP in cells preincubated with PRF at IC_0_ (Figure 8).

**Figure 9:**
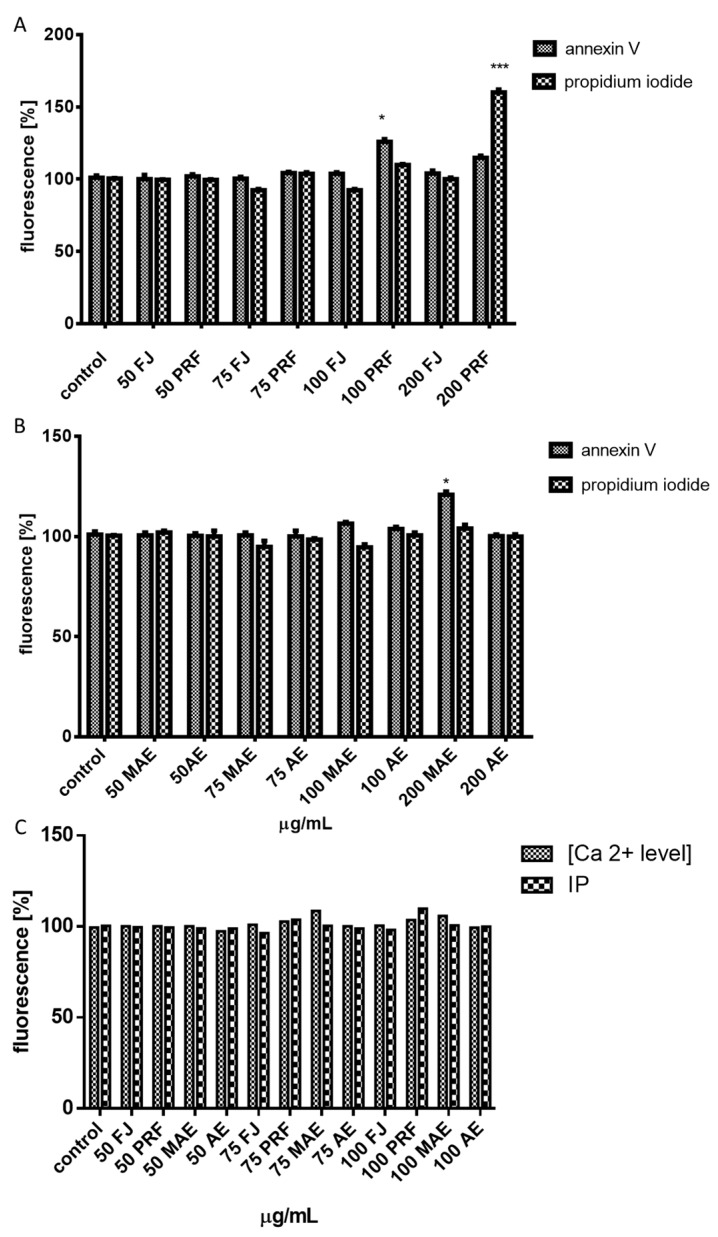
The influence of 24 h of exposure of juice (FJ) and PRF (phenolic rich fraction) (**A**), MAE (methanol-acetone extract) and AE (acetone extract) (**B**) on phosphatydylserine externalization on the outer membrane leaflet of apoptotic cells and membrane permeabilization detected with Annexin-V-FITC (fluorescein isothiocyanate) assay kit and propidium iodide staining (**A**,**B**); the extracts’ influence on intracellular calcium [Ca^2+^] level detected with Fluo-8 assay with simultaneous monitoring of possible cell membrane permeabilization based on propidium iodide (PI) DNA intercalation (**C**). Control cells were not exposed to any compound but the vehicle; values are means ± standard deviations from at least three independent experiments; statistical significance was calculated versus control cells (untreated) * *p* ≤ 0.05, *** *p* ≤ 0.001.

**Figure 10:**
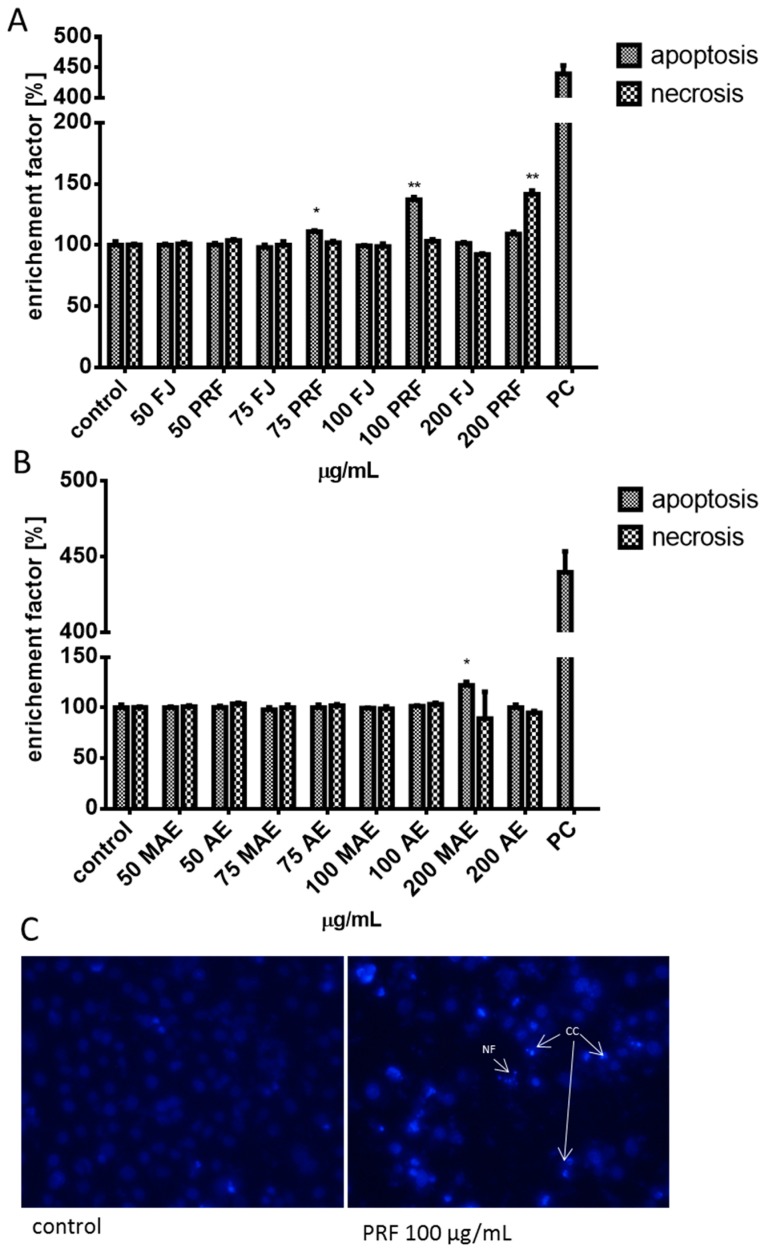
The influence of 24 h of exposure of juice (FJ) and PRF (phenolic rich fraction) (**A**), MAE (methanol-acetone extract) and AE (acetone extract) (**B**) on DNA fragmentation (late stage of apoptosis) analyzed by Cell Death Detection kit, PC—internal positive control of assay; nuclear morphology of DAPI-stained Caco-2 cells after 24 h of exposure to PRF at 100 μg/mL dosage. NF (nuclear fragmentation) and CC (chromatin condensation) in cells were observed (**C**) using fluorescence microscope (Nikon TS100 Eclipse, Japan), 200× magnification. Images are representative of one of four similar experiments. Control cells were not exposed to any compound but the vehicle; values are means ± standard deviations from at least three independent experiments; statistical significance was calculated versus control cells (untreated) * *p* ≤ 0.05, ** *p* ≤ 0.01.

Due to the observed decrease in intracellular ATP production and the intensive elevation of ROS we suspected that cellular death may be triggered. Thus, we carried out analysis of putative impact of extracts on apoptosis induction with detection of Annexin V binding to the externalized phosphatidylserine (PS) in the cell membrane (Figure 9A,B). The highest number of apoptotic cells positive for Annexin V staining was observed for 75–100 μg/mL of PRF (about 11%–35%). A prominent level of cells with propidium iodide stained nuclei (about 41%), specific for necrosis, was detected for PRF at 200 μg/mL. The absence of calcium ions’ intracellular flux in cells incubated in presence of 50–100 μg/mL of preparations confirmed the lack of their influence on permeability of the cellular membrane (Figure 9C), as well as the endoplasmic reticulum stress involvement in apoptosis induction. On the other hand, DNA fragmentation analysis of cytoplasmic mono-and oligonucleosomes confirmed the increase in apoptosis induction by comparable PRF extract dosages (Figure 10A,B). Caco-2 cells’ staining with DAPI showed the presence of chromatin condensation and nuclei fragmentation—these morphological changes are characteristic for late apoptosis (Figure 10C). Further investigation showed that cells treated with 200 μg/mL dosage of preparation induced predominantly necrotic death-type features due to the presence of cell released nucleosomes in culture medium, although it can result from the secondary necrosis that appears in apoptotic bodies not engulfed by neighboring cells. 

The observed *V. opulus* biological activity may be related with the proapoptotic potential of the main identified phenolic constituents: chlorogenic acid and flavanols. It was shown that chlorogenic acid and its microbial metabolites induced the S-phase cell-cycle arrest and activated caspase 3 at 500–1000 µM concentrations in Caco-2 cells [34]. Catechin, procyanidins B1 and B2 occurred as the next PRF constituents in terms of the amount, and these polyphenols, previously identified in grapes, Japanese quince fruit or cocoa, are potent caspase-dependent and independent apoptosis inducers in Caco-2 [35,36,37]. Importantly, the apoptosis induction was detected at relatively high dosages of extracts. In our studies we had no intention to stimulate cellular death, but we wanted to check cells’ response under acute influence of extracts. Where necrosis is recognized as inflammation due to damaged cells’ content leakage, we observed apoptosis induction by *V. opulus* would allow the avoidance of any inflammatory reaction and oxygen radical release from cells, being less destructive for neighboring cells [38]. To understand the preventive activity of *V. opulus* extracts against death induced by oxidative stress, there should be performed more detailed evaluation of specific markers connected with cellular death induction, such as caspases activation or appearance of specific proteins, i.e., t-Bid, cytochrome c, Bax, Bak [39]. Some of these proteins are known to be involved in necroptosis—an intrinsically regulated type of cell death mimicking features of apoptosis and necrosis. It was shown recently that green tea polyphenols were able to induce necroptosis in p53-deficient Hep3B cells through mitochondria-associated cell signaling pathways [40]. Due to these facts, identification of the detailed mechanism of *V. opulus* biological activity requires further investigation. 

### 3.6. Viburnum opulus Phenolics as Modulators of Fatty Acids Uptake

**Figure 11:**
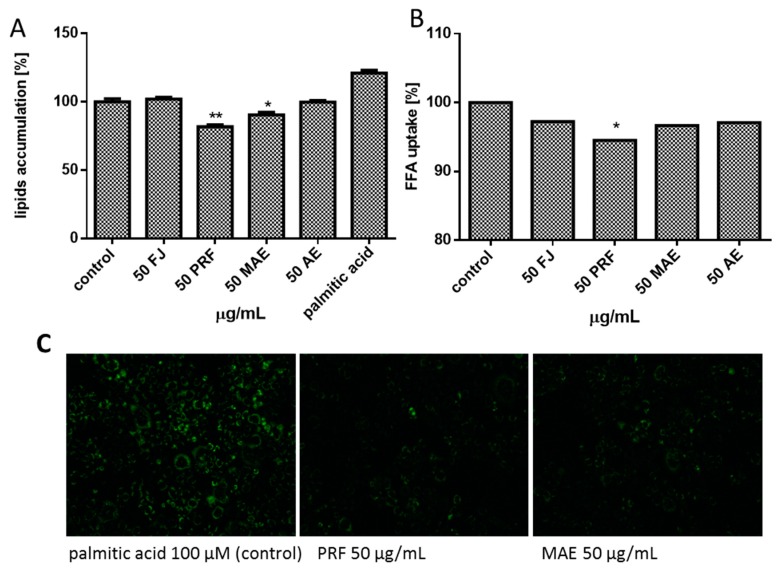
The influence of 24 h exposure of *V. opulus* juice (FJ), PRF (phenolic rich fraction), MAE (methanol-acetone extract) and AE (acetone extract) at 50 µg/mL on Caco-2 cells in the presence of 100 µM palmitic acid on the cellular lipid accumulation obtained with Nile red assay (**A**). The influence of 24 h exposure of extracts on fatty acid analogue TF2-C12 uptake measured with FFA-uptake assay (**B**). The influence of the PRF and MAE extracts on the accumulation of lipid droplets in Caco-2 cells stained with Nile red was observed by treating cells with the extracts for 24 h in the presence of palmitic acid (100 µM). High fluorescence in positive control cells after incubation with palmitic acid was observed. Fluorescence microscopy (Nikon TS100 Eclipse, Japan), 200× magnification. Images are representative of one of four similar experiments (**C**). Control cells were not exposed to any compound but the vehicle; values are means ± standard deviations from at least three independent experiments; statistical significance was calculated versus control cells (untreated) * *p* ≤ 0.05, ** *p* ≤ 0.01.

Overweight, obesity and type 2 diabetes are diet-related diseases strongly associated with the disturbance of homeostasis of the lipid metabolism. Because dietary fatty acids are absorbed in the digestive tract and further transported from intestine to the circulation, we checked the influence of *V. opulus* at IC_0_ on the level of lipids accumulation by cells co-incubated in the presence of palmitic acid (100 µM), which alone was not toxic to the cells after 24 h. It was found that the ability to decrease cellular lipid accumulation revealed PRF (17% inhibition compared to the untreated cells) and MAE (10%) (Figure 11A). The influence of preparations on the formation of intracellular lipid droplets (lipid bodies), organelles that serve as the storage of intracellular neutral lipids, stained with fluorescent Nile red, is presented in Figure 11C. The appearance of these organelles is connected with metabolic disorders [41], and it is visible that both extracts reduced the dimensions and number of accumulated lipid droplets. 

**Figure 12:**
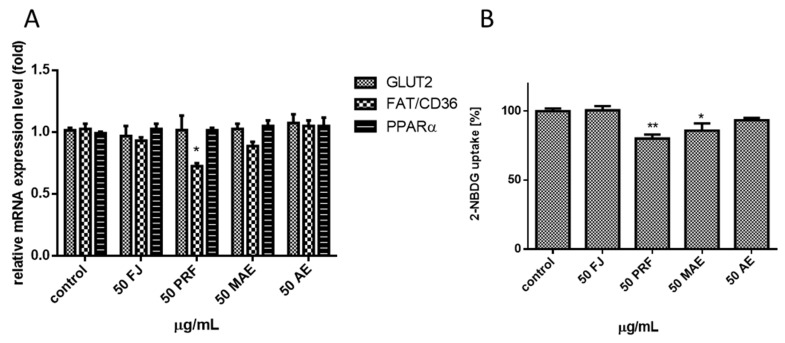
The influence of *V. opulus* juice (FJ) and PRF (phenolic rich fraction), MAE (methanol-acetone extract) and AE (acetone extract) on Caco-2 cells (24 h exposure, 50 µg/mL) on the expression of genes associated with glucose and free fatty acid uptake. The expression level of *GLUT2*, *FAT/CD36* and *PPARα* was quantified by real-time PCR and normalized using hypoxanthine phosphoribosyltransferase 1 (HPRT1) as a reference gene (**A**); the influence of extracts on fluorescent glucose analogue-2-NBDG—uptake (**B**). Control cells were not exposed to any compound but the vehicle; values are means ± standard deviations from at least three independent experiments; statistical significance was calculated versus control cells (untreated) * *p* ≤ 0.05, ** *p* ≤ 0.01.

To quantify the extracts’ effect on cellular FFA uptake. the level of incorporated fluorescent free fatty acid analogue TF2-C12 was measured. As indicated in Figure 11B, a noticeable decrease in fluorescence (ca. 5%) revealed only PRF. After cells incubation with all *V. opulus* extracts we did not observe the increase of intracellular Ca^2+^ concentration, where [Ca^2+^] elevation in turn may affect membrane permeability [42]. The obtained result allowed us to exclude the paracellular way of fatty acid transport by Caco-2 in favor of the mechanism using protein transporters. One of the main proteins involved in transmembrane movement of fatty acids is cluster of differentiation 36/fatty acid translocase (CD36/FAT), already classified as scavenger receptor B2 (SR-B2): CD36/SR-B2 [43]. It plays a relevant function in lipid metabolism: CD36 knockout mice revealed decreased fatty acid uptake in adipose tissue and reduced uptake of very long chain fatty acids in the intestine [44], however increased hepatic CD36/FAT expression has been observed in various pathologic conditions, such as insulin resistance and type 2 diabetes mellitus [45]. In order to investigate whether extracts influence FFA transport across the plasma membrane via influence on CD36/SR-B2, we checked *CD36* mRNA expression level. As shown in Figure 12A, cells’ incubation with PRF fraction resulted in ca. 25% decrease of *CD36* mRNA levels, suggesting a functional link between lowering the lipids’ accumulation and the level of FFA uptake. It is known that CD36/SR-B2 expression is regulated by agonists of the nuclear peroxisome proliferator-activated receptors (PPAR). Recent studies performed on 3T3L1 preadipocytes identified chlorogenic acid as PPARγ2 (20 µM) and PPARα (50 µM) agonist [46,47]. Our results revealed that *V. opulus* did not influence the expression of the gene encoding *PPARα* allowing us to suspect that other *V. opulus* polyphenolic compounds may act as PPAR or CD36/SR-B2 antagonist. The second hypothesis confirmed the PRF ability to decline both FFA uptake and MMP potential. A similar mechanism of action showed an established inhibitor of CD36/FAT translocase, succinimidyl oleate, which was able to lower mitochondrial membrane potential through the complex III inhibition [48]. However, the observed mechanism of phenolic activity might be much more complicated, because in protein mediated FFA metabolic trapping intracellular FA transport protein 4 (FATP4) and scavenger receptor B1 (SR-B1) are involved, which were not studied by us [49]. Because recent data performed on human subjects revealed the positive interaction between *CD36* variants and obesity on T2DM susceptibility [50], more detailed research on the influence of *V. opulus* phenolic compounds on lipids transport and metabolism is needed. 

### 3.7. Viburnum opulus Phenolic Compounds as Modulators of Glucose Uptake

The above results suggested that *V. opulus* can reduce the accumulation of lipids in Caco-2 cells, thus, to determine whether it has an effect on glucose uptake, after cells’ incubation with the polyphenols we measured the fluorescent analogue 2-NBDG incorporation. In Figure 12B it can be seen that incubation with extracts reduced glucose uptake at about by 7%–19% in comparison with the control, ranked as follows: PRF > MAE > AE. We did not observe the influence of FJ on 2-NBDG absorption. In observed glucose-uptake inhibiting *V. opulus* extracts were identified procyanidins. These polyphenols are able to permeate through the Caco-2 cell model and reduce glucose levels [51]. Other data shows that apple and strawberry extracts enriched with hydroxycinnamic acids and procyanidins were able to decrease glucose uptake and transport by human intestinal Caco-2 cells via inhibition of glucose transporter 2 (GLUT2) involved in bi-directional fluxes of glucose [13]. The GLUT2 protein is mainly involved in intestinal glucose transport across the enterocyte membrane. There are known many flavonoids able to attenuate GLUT2 activity and among them quercetin, as well as its glucoside precursor isoquercitrin, are mentioned [11]. Quercetin, which was identified in PRF and MAE extracts, was shown as a robust noncompetitive intestinal GLUT2 inhibitor not transported by glucose transporters [11]. We can assume that *Viburnum* phenolic constituents acted as GLUT2 proteins’ inhibitor. Observed uptake decrease may also result through the competition between different transporter agonists. Cyanidin-3-*O*-glucoside, one of the anthocyanins identified in *V. opulus*, could be transported through the Caco-2 cell monolayers in intact aglycone form [52]. Given that quercetin-3-*O*-glucoside is able to mitigate cyanidin-3-*O*-glucoside absorption we hypothesize that other *V. opulus* polyphenolic ingredients may compete for the same glucose transporter, block it and decrease glucose uptake. On the other hand, the detected decrease of glucose absorption may result through the lowering of GLUT2 protein level. Thus, in our further work we checked phenolic compounds’ effect on *GLUT2* gene expression. Although chlorogenic acid, the main phenolic constituent, was recently presented as a *GLUT2* positive expression regulator in the intestine of rats on a high fat diet [20], quantitative PCR analysis in our experiment revealed a lack of the extracts’ influence on *GLUT2* expression (Figure 12A). This could be in agreement with our supposition that *V. opulus* phenolics revealed a synergistic effect as GLUT2 protein inhibitors. However, despite these observations there could be other mechanisms explaining the obtained activity of these phytocompounds. Glucose transporters are embedded in membrane lipid bilayers, which are involved in signal transduction. The structure of membrane changes with fluctuation of glucose or lipids concentrations [53]. Modification of membrane lipids composition or lipids/proteins oxidation change the cellular membrane fluidity and permeability. These alterations influence function of transmembrane proteins, such as GLUTs or CD36/FAT, and, in turn, the uptake of glucose or FFA, respectively. Membrane rigidity is strongly influenced by ROS, which are responsible for lipid peroxidation and decrease of membrane fluidity leading to its damage [54]. Thus remarkably high antioxidant activity of *V. opulus* through scavenging of free radicals, inhibition of lipid oxidation and reduction of hydroperoxide formation may contribute to protection of membrane dynamic. Cellular membranes contain fluctuant microdomains known as lipid rafts. These rafts are linked with membrane proteins, like transporters, and change of raft deposition implicates transporter functionality. Nevertheless, besides ROS, polyphenols are also involved in lipid rafts shifting, and are able to bind to the lipids and form hydrogen bonds with the membrane. Depending on the chemical structure, they caused lipid membrane aggregation and rigidification: flavonoids majorly caused membrane aggregation, however, their glycosylation reduced that interactivity; stilbenes mediated membrane fluctuation, [55,56]. In general, the influence of polyphenols on membranes increases with the number of hydrophilic side chains (galloyl, hydroxyl, glucoside, gallate) and is connected with their antioxidant capacity. We suspect that glucose and FFA uptake attenuation is partly due to polyphenol–membrane interactions leading to the impairment of signal transduction or GLUT2 and CD36/FAT protein trafficking, although further studies are needed to support this hypothesis.

## 4. Conclusions

**Figure 13:**
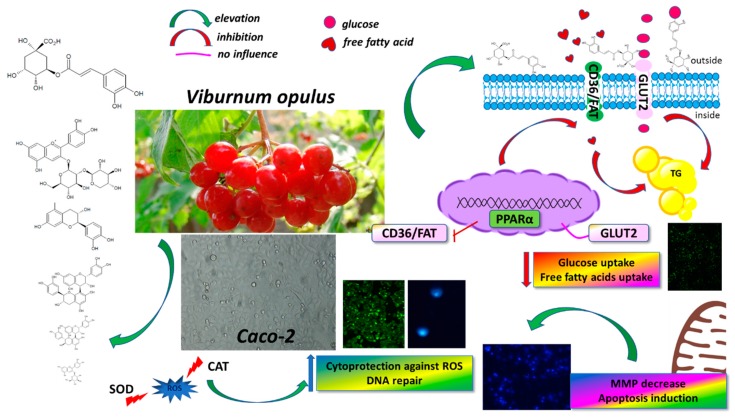
*Viburnum opulus* phenolic compounds as cytoprotective agents able to decrease free fatty acids and glucose uptake by Caco-2 cells—proposed mechanism of action. They possess cytoprotective activity against ROS generation, stimulate repair of DNA-damaged by mutagens and induce apoptotic type cell death. They are able to attenuate glucose and free fatty acid uptake through inhibition of FFA or glucose transporters. They influence the expression of *CD36/FAT* transporter, *FFA* or glucose transporters. CD36/FAT—cluster of differentiation 36/fatty acid translocase CD36/FAT; CAT—catalase; GLUT2—glucose transporter 2; PPARα—peroxisome proliferator activated receptor α; ROS—reactive oxygen species; SOD—superoxide dismutase; TG—triglycerides accumulated in adiposomes.

In this study, *Viburnum opulus* fruit was found to be a source of phenolic compounds identified as mainly chlorogenic acid, procyanidins, cyanidin glycosides and quercetin (Figure 13). Among studied extracts, phenolic rich fraction obtained from fruit juice (PRF) revealed the strongest activity to decrease uptake of FFA, glucose and accumulation of lipid droplets in Caco-2 cells without affecting their viability. Thus *V. opulus* phenolics might provide a dietary mechanism for regulating and delaying the rate of glucose and fatty acids absorption by intestinal cells. Observed uptake attenuation was followed by decrease of the *CD36/FAT* gene expression, without influence on the *GLUT2* and *PPARα* levels. We suspect that *V. opulus* phenolics may act as transporter inhibitors or modulate cellular membrane dynamics, although this hypothesis requires further, more-detailed studies. Still, at the same dosage extract protected cells against t-BOOH generated oxidative stress and DNA-damage induced by mutagens. Furthermore, acute incubation with the highest concentration of compounds induced apoptosis instead of necrosis, which in turn diminishes inflammatory response due to the leakage decrease of intracellular components from necrotic damaged cells. To the best of our knowledge, this is a pioneering study to show *V. opulus* phenolic compounds as cytoprotective agents able to decrease free fatty acids and glucose uptake by Caco-2 cells. 

In summary, we proposed a preliminary mechanism accounting for activity of *Viburnum opulus* phenolic compounds, however, these properties need further molecular evaluation of the beneficial effects of extracts after in vitro digestion or obtained after its incubation with gut microflora. 

## Figures and Tables

**Table 1:** Individual phenolic compounds content in *Viburnum opulus* samples.

Hydroxybenzoic Acid (HBA)	R_t_ [min]	λ_max_ [nm]	FJ	MAE	AE	PRF
			mg/g of Juice	mg/g of Extract		
Gallic acid	1.92	282.7	n.d.	0.21 ± 0.00 *^a^*	0.40 ± 0.00 *^b^*	n.d.
Flavanols			1.21 ± 0.01	22.84 ± 0.05	15.46 ± 0.22	100.74 ± 0.10
Procyanidin B1	4.50	278.7	0.31 ± 0.00 *^b^*	3.28 ± 0.03 *^c^*	2.46 ± 0.01 *^a^*	29.76 ± 0.05 *^d^*
(+)-Catechin	5.19	278.7	0.68 ± 0.01 *^b^*	9.71 ± 0.01 *^c^*	5.70 ± 0.00 *^a^*	53.18 ± 0.03 *^d^*
Procyanidin B2	5.98	278.7	0.22 ± 0.00 *^a^*	5.44 ± 0.02 *^c^*	3.77 ± 0.03 *^b^*	17.79 ± 0.02 *^d^*
(−)-Epicatechin	6.85	277.7	n.d.	4.42 ± 0.02 *^b^*	3.53 ± 0.18 *^a^*	n.d.
Hydroxycinnamic Acids (HCA)			7.97 ± 0.02	96.37 ± 0.14	84.27 ± 0.26	645.85 ± 2.00
Neochlorogenic acid	3.94	323.7	n.d.	0.16 ± 0.00 *^b^*	0.15 ± 0.00 *^a^*	0,36 ± 0.00 *^c^*
Chlorogenic acid	5.55	325.7	7.97 ± 0.02 *^a^*	96.22 ± 0.14 *^c^*	84.31 ± 0.26 *^b^*	645.49 ± 2.00
Flavonols			0.01 ± 0.00	0.54 ± 0.00	0.48 ± 0.00	2.35 ± 0.00
Rutin	9.66	353.7	0.01 ± 0.00 *^b^*	n.d.	0.12 ± 0.00 *^a^*	1.09 ± 0.00 *^c^*
Isorhamnetin	9.93	353.7	0.002 ± 0.000 *^a^*	n.d.	n.d.	0.12 ± 0.00 *^b^*
Isorhamnetin 3-*O*-rutinoside	11.20	353.7	0.01 ± 0.00 *^a^*	0.26 ± 0.00*^c^*	0.18 ± 0.00 *^b^*	0.84 ± 0.00 *^d^*
Quercetin	11.55	352.7	n.d.	0.28 ± 0.00 *^b^*	0.17 ± 0.00 *^a^*	0.30 ± 0.00 *^c^*
Anthocyanins			1.12 ± 0.00	2.07 ± 0.01	1.45 ± 0.00	78.06 ± 0.68
Cyanidin-3-*O*-sambubioside	6.83	516.7	0.85 ± 0.00	0.74 ± 0.00	0.56 ± 0.00	55.78 ± 0.69
Cyanidin-3-*O*-glucoside	7.03	515.7	0.20 ± 0.01	0.86 ± 0.00	0.58 ± 0.00	17.08 ± 0.01
Cyanidin-3-*O*-rutinoside	7.36	518.7	0.06 ± 0.00	0.47 ± 0.00	0.31 ± 0.00	5.21 ± 0.01
Phenolics Total			10.32 ± 0.03	121.83 ± 0.19	101.67 ± 0.41	827.00 ± 1.68

n.d.—not detected; FJ—fresh juice, MAE—methanol-acetone extract from pomace; AE—acetone extract from pomace; PRF—phenolic rich fraction from FJ. Results are expressed as a mean ± standard deviation (n = 3). The values expressed with the different superscript letter (*a, b, c*) within the same column differ significantly (one-way ANOVA and Duncan’s test, *p* ≤ 0.05).

**Table 2:** Total phenolic content of *Viburnum opulus* samples and their antioxidant capacity estimated by ABTS and ORAC methods.

Sample	Total Phenolics	Antioxidant Capacity	
		ABTS	ORAC
	mg GAE/g	µmol TE/g	
Methanol-Acetone Extract (MAE)	143.77 ± 4.0 ^b^	1230.81 ± 26.1 ^b^	2388.58 ± 150.5^c^
Acetone Extract (AE)	137.80 ± 5.6 ^b^	1227.73 ± 32.0 ^b^	2092.65 ± 48.3 ^b^
Phenolic Rich Fraction (PRF)	361.52 ± 13.3 ^c^	2619.59 ± 123.1^c^	7810.29 ± 342.3^d^
Fresh Juice (FJ)	5.98 ± 0.3 ^a^	38.36 ± 2.7 ^a^	103.50 ± 6.4 ^a^

Results are expressed as a mean ± standard deviation (n ≥ 4); ^a–d^—the same letters within one analyzed parameter (column) denote no statistically significant differences at *p* ≤ 0.05.

**Table 3:** The influence of *Viburnum opulus* fractions on Caco-2 cells viability presented as IC_0_ and IC_50_ values.

***V. opulus* Preparation**	**IC_0_ [µg/mL]**	**IC_50_ [µg/mL]**		**Cells Viability [%] ^1^**
			**t-BOOH 500 μM**	79.50 ± 3.85
FJ	75.0	450.0		95.64 ± 8.78
PRF	50.0	250.0		96.12 ± 10.12
MAE	50.0	275.0		91.70 ± 8.31
AE	100	400.0		90.50 ± 3.02

The influence of tested compounds was measured with PrestoBlue assay; ^1^ The influence of cells’ preincubation with maximal nontoxic extracts concentrations (IC_0_) for 24 h against oxidative stress induced by 500 μM t-BOOH (2 h) on metabolic activity.

## References

[1] Cory H., Passarelli S., Szeto J., Tamez M., Mattei J. (2018). The Role of Polyphenols in Human Health and Food Systems: A Mini-Review. Front. Nutr..

[2] Eken A., Yücel O., İpek İ., Ayşe B., Endİrlİk B.Ü. (2017). An Investigation on Protective Effect of *Viburnum opulus* L. Fruit Extract Against Ischemia/Reperfusion-Induced Oxidative Stress after lung transplantation in rats. Kafkas Üniversitesi Veteriner Fakültesi Dergisi.

[3] Perova I.B., Zhogova A.A., Cherkashin A.V., Éller K.I., Ramenskaya G.V. (2014). Biologically Active Substances From European Guelder Berry Fruits. Pharm. Chem. J..

[4] Moldovan B., David L., Chişbora C., Cimpoiu C. (2012). Degradation Kinetics of Anthocyanins from European Cranberrybush (*Viburnum opulus* L.) Fruit Extracts. Effects of Temperature, pH and Storage Solvent. Molecules.

[5] Kızılay O.N., Ülker F., Çelik V., Özdemir T., Çakmak Ö., Can E. (2019). The evaluation of the effectiveness of Gilaburu (*Viburnum opulus* L.) extract in the medical expulsive treatment of distal ureteral stones The evaluation of the effectiveness of Gilaburu (*Viburnum opulus* L.) extract in the medical expulsive treatment of distal ureteral stones. Turk. J. Urol..

[6] Ceylan D., Aksoy A., Ertekin T., Yay A.H., Nisari M., Karatoprak G.Ş., Ülger H. (2018). The effects of gilaburu (Viburnum opulus) juice on experimentally induced Ehrlich ascites tumor in mice. J. Cancer Res. Ther..

[7] Kozlowska W., Wagner C., Moore E.M., Matkowski A. (2018). Botanical Provenance of Traditional Medicines From Carpathian Mountains at the Ukrainian-Polish Border. Front. Pharmacol..

[8] Zakłos-Szyda M., Majewska I., Redzynia M., Koziołkiewicz M. (2015). Antidiabetic Effect of Polyphenolic Extracts from Selected Edible Plants as α-Amylase, α-Glucosidase and PTP1B Inhibitors, and β Pancreatic Cells Cytoprotective Agents—A Comparative Study. Curr. Top. Med. Chem..

[9] Mihaylova D., Popova A., Alexieva I., Krastanov A., Lante A. (2018). Polyphenols as Suitable Control for Obesity and Diabetes. Open Biotech. J..

[10] Farrell T.L., Ellam S.L., Forrelli T., Williamson G. (2013). Attenuation of glucose transport across Caco-2 cell monolayers by a polyphenol-rich herbal extract: Interactions with SGLT1 and GLUT2 transporters. Biofactors.

[11] Kwon O., Eck P., Chen S., Corpe C.P., Lee J.H., Kruhlak M., Levine M. (2007). Inhibition of the intestinal glucose transporter GLUT2 by flavonoids. FASEB J..

[12] Schreck K., Melzig M.F. (2018). Intestinal Saturated Long-Chain Fatty Acid, Glucose and Fructose Transporters and Their Inhibition by Natural Plant Extracts in Caco-2 Cells. Molecules.

[13] Manzano S., Williamson G. (2010). Polyphenols and phenolic acids from strawberry and apple decrease glucose uptake and transport by human intestinal Caco-2 cells. Mol. Nutr. Food Res..

[14] Stremmel W., Staffer S., Wannhoff A., Pathil A. (2017). The overall fatty acid absorption controlled by basolateral chylomicron excretion under regulation of p-JNK1. Biochim. Biophys. Acta-Mol. Cell Biol. Lipids.

[15] Podsędek M., Majewska A., Redzynia I., Sosnowska M., Koziołkiewicz D. (2014). In vitro inhibitory effect on digestive enzymes and antioxidant potential of commonly consumed fruits. J. Agric. Food Chem..

[16] Budryn G., Zakłos-Szyda M., Zaczyńska D., Żyżelewicz D., Grzelczyk J., Zduńczyk Z., Juśkiewicz J. (2017). Green and roasted coffee extracts as antioxidants in βTC3 cells with induced oxidative stress and lipid accumulation inhibitors in 3T3L1 cells, and their bioactivity in rats fed high fat diet. Eur. Food Res. Technol..

[17] Zakłos-Szyda M., Pawlik N. (2018). Japanese quince (*Chaenomeles japonica* L.) fruit polyphenolic extract modulates carbohydrate metabolism in HepG2 cells via AMP-activated protein kinase. Acta Biochim. Pol..

[18] Hogan A.M., Swaminathan V., Pallegar N.K., Christian S.L. (2016). Nile Red and 2-NBDG Are Incompatible for the Simultaneous Detection of Lipid and Glucose Accumulation. Int. J. Spectrosc..

[19] Nowak A., Klewicki R., Lipin L. (2017). Ellagitannins from *Rubus idaeus* L. Exert Geno—And Cytotoxic Effects against Human Colon Adenocarcinoma Cell Line Caco-2. J. Agric. Food Chem..

[20] Peng B.J., Zhu Q., Zhong Y.L., Xu S.H., Wang Z. (2015). Chlorogenic Acid Maintains Glucose Homeostasis through Modulating the Expression of SGLT-1, GLUT-2, and PLG in Different Intestinal Segments of Sprague-Dawley Rats Fed a High-Fat Diet. Biomed. Environ. Sci..

[21] Velioglu Y.S., Ekici L., Poyrazoglu E.S. (2006). Original article Phenolic composition of European cranberrybush (*Viburnum opulus* L.) berries and astringency removal of its commercial juice. Int. J. Food Sci. Technol..

[22] Karaçelik P., Küçük A.A., Iskefiyeli M., Aydemir Z., de Smet S., Miserez S., Sandra B. (2015). Antioxidant components of *Viburnum opulus* L. determined by on-line HPLC–UV–ABTS radical scavenging and LC–UV–ESI-MS methods. Food Chem..

[23] Kraujalyte V., Rimantas P., Pukalskas A., Laima C. (2013). Antioxidant properties and polyphenolic compositions of fruits from different European cranberrybush (*Viburnum opulus* L.) genotypes. Food Chem..

[24] De Freitas V., Faria A., Pestana D., Azevedo J., Azevedo I., Mateus N. (2009). Absorption of anthocyanins through intestinal epithelial cells—Putative involvement of GLUT2. Mol. Nutr. Food Res..

[25] Hajiaghaalipour F., Khalilpourfarshbafi M., Arya A. (2015). Modulation of Glucose Transporter Protein by Dietary Flavonoids in Type 2 Diabetes Mellitus. Int. J. Biol. Sci..

[26] Catarino T.A., Gonc P. (2013). The effect of oxidative stress upon the intestinal epithelial uptake of butyrate. Eur. J. Pharmacol..

[27] Margina D., Gradinaru D., Manda G., Neagoe I., Ilie M. (2013). Membranar effects exerted in vitro by polyphenols—Quercetin, epigallocatechin gallate and curcumin—On HUVEC and Jurkat cells, relevant for diabetes mellitus. Food Chem. Toxicol..

[28] Amararathna M., Johnston M.R., Rupasinghe H.P.V. (2016). Plant Polyphenols as Chemopreventive Agents for Lung Cancer. Int. J. Mol. Sci..

[29] Goszcz K., Duthie G.G., Stewart D., Leslie S.J., Megson I.L. (2017). Bioactive polyphenols and cardiovascular disease: Chemical antagonists, pharmacological agents or xenobiotics that drive an adaptive response?. Br. J. Pharmacol..

[30] Liang N., Dupuis J.H., Yada R.Y., Kitts D.D. (2019). Chlorogenic acid isomers directly interact with Keap 1-Nrf2 signaling in Caco-2 cells. Mol. Cell. Biochem..

[31] Bonarska-kujawa H.P.D. (2014). Effect of chlorogenic acid on the phase transition in phospholipid and phospholipid/cholesterol membranes. J. Thermal Anal. Calorim..

[32] Liang N., Kitts D.D. (2018). Chlorogenic Acid (CGA) Isomers Alleviate Interleukin 8 (IL-8) Production in Caco-2 Cells by Decreasing Phosphorylation of p38 and Increasing Cell Integrity. Int. J. Mol. Sci..

[33] Wang J., Li J., Liu J., Xu M., Tong X., Wang J. (2016). Chlorogenic acid prevents isoproterenol-induced DNA damage in vascular smooth muscle cells. Mol. Med. Rep..

[34] Ekbatan S.S., Li X., Ghorbani M., Azadi B. (2018). Chlorogenic Acid and Its Microbial Metabolites Exert Anti-Proliferative Effects, S-Phase Cell-Cycle Arrest and Apoptosis in Human Colon Cancer Caco-2 Cells. Int. J. Mol. Sci..

[35] Cheah K.Y., Howarth G.S., Bindon K.A., Kennedy J.A., Bastian S.E.P. (2014). Low molecular weight procyanidins from grape seeds enhance the impact of 5-Fluorouracil chemotherapy on Caco-2 human colon cancer cells. PLoS ONE.

[36] Gorlach S., Wagner W., Podsędek A., Szewczyk K., Koziołkiewicz M., Dastych J. (2011). Procyanidins from Japanese Quince (Chaenomeles japonica) fruit induce apoptosis in human colon cancer Caco-2 cells in a degree of polymerization-dependent manner. Nutr. Cancer.

[37] Rodríguez-ramiro I., Ramos S., Bravo L., Goya L., Martín M.Á. (2011). Procyanidin B2 and a cocoa polyphenolic extract inhibit acrylamide-induced apoptosis in human Caco-2 cells by preventing oxidative stress and activation of JNK pathway. J. Nutr. Biochem..

[38] Pandey M.K., Gupta S.C., Nabavizadeh A., Aggarwal B.B. (2017). Seminars in Cancer Biology Regulation of cell signaling pathways by dietary agents for cancer prevention and treatment. Semin. Cancer Biol..

[39] Dhuriya Y.K., Sharma D. (2018). Necroptosis: A regulated inflammatory mode of cell death. J. Neuroinflamm..

[40] Lin W., Tongyi S. (2014). Role of Bax/Bcl-2 family members in green tea polyphenol induced necroptosis of p53-deficient Hep3B cells. Tumor Biol..

[41] Fam T., Klymchenko A., Collot M. (2018). Recent Advances in Fluorescent Probes for Lipid Droplets. Materials.

[42] Rohm B., Riedel A., Ley J.P., Widder S. (2015). Function activation and increase acetyl-coenzyme A. Food Funct..

[43] Glatz J.F.C., Luiken J.J.F.P. (2017). Biochimie From fat to FAT (CD36/SR-B2): Understanding the regulation of cellular fatty acid uptake. Biochimie.

[44] Schneider H., Staudacher S., Poppelreuther M., Stremmel W., Ehehalt R., Füllekrug J. (2014). Protein mediated fatty acid uptake: Synergy between CD36/FAT-facilitated transport and acyl-CoA synthetase-driven metabolism. Arch. Biochem. Biophys..

[45] Perona J.S. (2017). Biochimica et Biophysica Acta Membrane lipid alterations in the metabolic syndrome and the role of dietary oils. BBA-Biomembr..

[46] Sancheza M.B., Miranda-Pereza E., Verjanb J.C.G., Barreraa M.D.F., Perez-Ramosc J., Alarcon-Aguilaret F.J. (2017). Potential of the chlorogenic acid as multitarget agent: Insulin-secretagogue and PPAR α/γ dual agonist. Biomed. Pharmacother..

[47] Pang Y., Zhu Q., Kang J., Liu M., Wang Z. (2018). Chlorogenic Acid Functions as a Novel Agonist of PPAR γ 2 during the Differentiation of Mouse 3T3-L1 Preadipocytes. BioMed Res. Int..

[48] Vrbacky M. (2010). Succinimidyl oleate, established inhibitor of CD36/FAT translocase inhibits complex III of mitochondrial respiratory chain. Biochem. Biophys. Res. Commun..

[49] Abumrad N.A., Cifarelli V. (2018). Intestinal CD36 and Other Key Proteins of Lipid Utilization: Role in Absorption and Gut Homeostasis. Compr. Physiol..

[50] Zhang D., Zhang R., Liu Y., Sun X., Yin Z., Li H. (2018). CD36 gene variants is associated with type 2 diabetes mellitus through the interaction of obesity in rural Chinese adults. Gene.

[51] Hemmersbach S., Brauer S.S., Hu S., Galla H., Humpf H. (2013). Transepithelial Permeability Studies of Flavan-3-ol-C-glucosides and Procyanidin Dimers and Trimers across the Caco-2 Cell Monolayer. J. Agric. Food Chem..

[52] Zou T., Feng D., Song G., Li H., Tang H., Ling W. (2014). The Role of Sodium-Dependent Glucose Transporter 1 and Glucose Transporter 2 in the Absorption of Cyanidin-3-O-β-Glucoside in Caco-2 Cells. Nutrients.

[53] Hresko R.C., Kraft T.E., Quigley A., Carpenter E.P., Hruz P.W. (2016). Mammalian Glucose Transporter Activity Is Dependent upon Anionic and Conical Phospholipids. J. Biol. Chem..

[54] De C., Palacio J.R., Martínez P., Morros A. (2013). Biochimica et Biophysica Acta Effect of oxidative stress on plasma membrane fluidity of THP-1 induced macrophages. BBA-Biomembr..

[55] Phan H.T.T., Yoda T., Chahal B., Morita M., Takagi M., Vestergaard C. (2014). Biochimica et Biophysica Acta Structure-dependent interactions of polyphenols with a biomimetic membrane system. BBA-Biomembr..

[56] Tsuchiya H. (2015). Membrane Interactions of Phytochemicals as Their Molecular Mechanism Applicable to the Discovery of Drug Leads from Plants. Molecules.

